# A small molecule chaperone rescues the stability and activity of a cancer‐associated variant of NAD(P)H:quinone oxidoreductase 1 *in vitro*

**DOI:** 10.1002/1873-3468.13636

**Published:** 2019-10-30

**Authors:** Emilia Strandback, Wolf‐Dieter Lienhart, Altijana Hromic‐Jahjefendic, Benjamin Bourgeois, Anja Högler, Daniel Waltenstorfer, Andreas Winkler, Klaus Zangger, Tobias Madl, Karl Gruber, Peter Macheroux

**Affiliations:** ^1^ Institute of Biochemistry Graz University of Technology Austria; ^2^ Institute of Molecular Biosciences University of Graz Austria; ^3^ Department of Genetics and Bioengineering Faculty of Engineering and Natural Sciences International University of Sarajevo Sarajevo Bosnia and Herzegovina; ^4^ Gottfried Schatz Research Center for Cell Signaling, Metabolism and Aging, Molecular Biology and Biochemistry Medical University of Graz Austria; ^5^ Institute of Chemistry University of Graz Austria; ^6^ BioTechMed‐Graz Austria

**Keywords:** cancer, chemical chaperone, chemotherapeutics, quinone, single‐nucleotide polymorphism

## Abstract

NAD(P)H:quinone oxidoreductase 1 (NQO1) is a human FAD‐dependent enzyme that plays a crucial role in the antioxidant defense system. A naturally occurring single‐nucleotide polymorphism (NQO1*2) in the *NQO1* gene leads to an amino acid substitution (P187S), which severely compromises the activity and stability of the enzyme. The NQO1*2 genotype has been linked to a higher risk for several types of cancer and poor survival rate after anthracycline‐based chemotherapy. In this study, we show that a small molecular chaperone (N‐(2‐bromophenyl)pyrrolidine‐1‐sulfonamide) repopulates the native wild‐type conformation. As a consequence of the stabilizing effect, the enzymatic activity of the P187S variant protein is strongly improved in the presence of the molecular chaperone *in vitro*.

## Graphical abstract

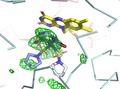

## Abbreviations

**BPPSA**, N‐(2‐bromophenyl)pyrrolidine‐1‐sulfonamide

**HDX‐MS**, hydrogen‐deuterium exchange coupled to mass spectrometry

**HSQC**, heteronuclear single quantum coherence

**ITC**, isothermal titration calorimetry

**NQO1**, NAD(P)H:quinone oxidoreductase 1

**PDB**, protein data bank

**SAXS**, small‐angle X‐ray scattering

**SNP**, single‐nucleotide polymorphism

**WT**, wild‐type

NAD(P)H:quinone oxidoreductase 1 (NQO1; http://www.chem.qmul.ac.uk/iubmb/enzyme/EC1/6/99/2.html) is a human FAD‐dependent enzyme catalyzing the two‐electron reduction of quinones to hydroquinones. NQO1, for instance, plays an important role in the antioxidant defense system where it lowers the quinone levels and thereby prevents the formation of reactive oxygen species 1, 2. Furthermore, it binds to the 20*S* proteasome, thus rescuing several tumor suppressors like p33^ING1b^, p53, and p73 from proteasomal degradation 2, 3. Due to the broad substrate scope, NQO1 can activate chemotherapeutic prodrugs, such as mitomycin C and β‐lapachone, and since the level of NQO1 is increased in several tumors, the quinone‐based chemotherapeutic activity will be specifically observed on the tumor cells 4, 5, 6.

A naturally occurring single‐nucleotide polymorphism (SNP) (NQO1*2) in the *NQO1* gene (C609T, on chromosome 16q22.1) results in the replacement of proline 187 with serine (P187S) in the amino acid sequence 7. The distribution of the homozygous NQO1*2 in the human population varies between 2% and 20% depending on the ethnic background and is most common in Asian, Mexican Hispanic, and Native American populations 8, 9. The NQO1*2 genotype is associated with an increased toxicity of benzene and higher risk for several types of cancers including lung, colon, breast, and leukemia 10, 11, 12. Additionally, an adverse breast cancer outcome has been reported after anthracycline‐based chemotherapy, with a survival rate of 17% for patients with the NQO1*2 genotype compared to 75% for other genotypes 11.

In a previous study, we have demonstrated that the NQO1 P187S variant is unstable in solution, although its crystallographic structure is virtually identical to the wild‐type protein 13. This instability is apparently caused by proline to serine substitution, which weakens the interaction between the core and C‐terminal domain of the P187S variant protein. It was also shown that the cofactor affinity and thereby also the activity are affected in the P187S variant protein as a consequence of the structural instability 13. This observation led to the idea that binding of a small molecule may reduce the conformational flexibility of the protein and thereby may exert a beneficial effect on the stability and activity of the P187S variant protein. A similar approach has already been described by Medina‐Carmona *et al*., where the strong competitive inhibitor dicoumarol, first described by Hosoda *et al*., was shown to improve the stability of the P187S variant protein 14, 15. Since dicoumarol is a strong inhibitor, this will, however, not be of any benefit when it comes to enzyme activity. There are also other studies available where chemical chaperones have been used. This includes, for example, migalastat that is a pharmacological chaperone used to treat Fabry disease 16, 17, 18 and studies of alpha‐1 antitrypsin deficiency 19, 20.

In the present study, we report on the beneficial effect of a ligand that binds to and stabilizes the NQO1 P187S variant. We have employed biophysical methods to demonstrate that this ligand, N‐(2‐bromophenyl)pyrrolidine‐1‐sulfonamide (BPPSA), induces structural properties very similar to that of the wild‐type protein. Moreover, it could be shown that, as a result of the stabilizing effect, BPPSA improves the enzymatic activity of the NQO1 P187S variant *in vitro*.

## Experimental procedures

### Reagents

All chemicals and reagents were of highest quality available either from Sigma‐Aldrich (St. Louis, MO, USA), Carl Roth GmbH (Karlsruhe, Germany), or Thermo Fisher Scientific (Waltham, MA, USA) unless otherwise mentioned. Purification columns were obtained from GE Healthcare (Little Chalfont, UK). Trypsin was from Promega (Madison, WI, USA). Labeled ammonium sulfate was from CortecNet (Voisins‐Le‐Bretonneux, France). The investigated ligand BPPSA was purchased from Enamine LLC (Monmouth Junction, NJ, USA).

### Molecular cloning, heterologous protein expression, and purification of NQO1

The cloning of NQO1 and the generation of the NQO1 P187S variant have been described previously 13. Briefly, the *NQO1* gene optimized for expression in *Escherichia coli* was obtained from GeneArt (Carlsbad, CA, USA) and cloned into a pET28a vector from Merck (Darmstadt, Germany). The NQO1 P187S variant was generated with the Quick Change II XL Site‐Directed Mutagenesis Kit from Agilent (Santa Clara, CA, USA) by the use of gene‐specific primers from Eurofins (Luxembourg). The protein expression and purification were performed according to the procedure described by Lienhart *et al*. 13. In brief, the protein expression was carried out in lysogeny broth (bacto‐tryptone 10 g·L^−1^, bacto yeast extract 5 g·L^−1^, NaCl 5 g·L^−1^) containing 50 μg·L^−1^ kanamycin. The main culture was inoculated with 5% of an overnight culture and incubated at 37 °C at 140 r.p.m. in shake flasks in a HT Multitron Standard shaking system (Infors AG, Basel, Switzerland) until an OD_600_ of around 0.8 was reached, and the cultures were then induced with 0.5 mm isopropyl‐β‐D‐thiogalactoside and grown for 16 h at 25 °C. Cells were harvested by centrifugation at 4420 ***g*** at 4 °C for 10 min, resuspended in 1% saline solution, and thereafter centrifuged again at 4570 ***g*** for 45 min. The cell pellets were then resuspended in 2 mL·g^−1^ pellet of lysis buffer (50 mm HEPES, 150 mm NaCl, 10 mm imidazole, pH 7.0) and 1 mg FAD, 10 μL protease inhibitor cocktail for histidine‐tagged proteins (Sigma‐Aldrich), 1 mm phenylmethylsulfonyl fluoride dissolved in dimethylsulfoxide, and 20 μg·mL^−1^ lysozyme were added to 25 mL of the cell suspension. Next, the cells were disrupted by sonication in a Sonopuls glass rosette cell RZ (Bandelin, Berlin, Germany) on ice with a Labsonic L instrument from Braun Biotech. International (Berlin, Germany) with 120 W for 6 × 3 min and 3‐min pause in between each cycle. The cell debris was removed by centrifugation at 38 470 ***g*** at 4 °C for 1 h. The supernatant was then loaded onto a 5 mL HisTrap FF column equilibrated with lysis buffer, whereafter the column was washed with wash buffer (50 mm HEPES, 150 mm NaCl, 50 mm imidazole, pH 7.0). Proteins were finally eluted with elution buffer (50 mm HEPES, 150 mm NaCl, 300 mm imidazole, pH 7.0). The protein‐containing fractions were combined and concentrated by using centrifugal filter units with 10 kDa cutoff from Pall Life Sciences (Ann Arbor, MI, USA). The concentrated protein was further purified by size‐exclusion chromatography on a HiLoad 16/60 Superdex 200 prep grade column equilibrated with 50 mm HEPES and 150 mm NaCl, pH 7.0, at 4 °C. The column was connected to an ÄKTA FPLC system (GE Healthcare). The purity of the protein‐containing fractions was checked using SDS/PAGE, and the pure fractions were then pooled and concentrated by using centrifugal filter units and finally rebuffered with a PD‐10 desalting column to 50 mm HEPES, pH 7.0. The protein was then shock frozen and stored at −80 °C.

### Virtual screening

Potentially stabilizing compounds were found by *in silico* docking using DOCK blaster (http://blaster.docking.org, University of California, SF, USA) and refinement 21. Dockings were performed using the available structure of the NQO1 P187S variant (PDB code http://www.rcsb.org/pdb/search/structidSearch.do?structureId=4CET). In order to search for possible lead compounds, the complete ZINC database (http://zinc.docking.org, University of California, SF, USA) with commercially available lead compounds was used. Based on the screening results, eight compounds were selected for the initial experimental screening.

### Thermal shift assay

The melting point determinations were performed as described by Forneris *et al*. and Ericsson *et al*. 22, 23. Briefly, 150 μm NQO1 WT or P187S variant in 50 mm HEPES at pH 7.0 with or without BPPSA at a concentration of 2 mm were mixed in a white 96‐well RT‐PCR plate (Bio‐Rad Laboratories, Hercules, CA, USA) sealed with optical‐quality sealing tape (Bio‐Rad Laboratories). The plate was heated from 20 to 95 °C, with 0.5 °C steps, in a CFX Connect™ Real‐Time PCR Detection System from Bio‐Rad Laboratories, and the change in fluorescence due to the release of the FAD cofactor upon unfolding of the protein was detected. The thermal stability was analyzed using the cfx manager 3.0 software from Bio‐Rad Laboratories.

### UV/Vis absorption difference titration

The difference absorption spectra were recorded with a Specord 200 plus spectrophotometer from Analytik Jena (Jena, Germany) at 25 °C in tandem cuvettes from Hellma Analytic (Müllheim, Germany) as described by Lienhart *et al*. 13. For the measurement, 800 μL of NQO1 WT or NQO1 P187S variant in 50 mm HEPES, pH 7.0, at a concentration of 40 μm in the measurement cell, was titrated with BPPSA (in 2‐μL steps from a 2 mm stock, dissolved in 50% v/v EtOH). The same volume of compound was added to the buffer chamber in the reference cuvette at the same time as the same volume of 50% EtOH in buffer was added to the protein chamber in the reference cuvette in order to always keep the dilution the same in all chambers. For the analysis, the sum of the absorption values at 444 and 496 nm in the case of NQO1 WT and 440 and 491 nm in the case of the NQO1 P187S variant were plotted against the concentration of BPPSA.

### Isothermal titration calorimetry

Isothermal titration calorimetry (ITC) measurements were performed using a PEAQ‐ITC (Malvern Instruments Ltd., Malvern, Worcestershire, UK). In the experiments, 380 μm NQO1 WT or P187S variant were used with 23 and 13 μm BPPSA, respectively. All samples were prepared in 50 mm HEPES at pH 7.0 with 1% DMSO in the final samples. The experiments were performed at 25 °C, with a stirring speed of 500 r.p.m. One experiment consisted of 13–14 injections with a volume of 2.8 μL and an injection duration of 5.6 s (including the first injection with a volume of 0.4 μL), and the spacing used was 250 s. Control experiments were performed by titrating 380 μm NQO1 WT or NQO1 P187S into a buffer with 1% DMSO. The analysis was done with the microcal peaq‐itc Analysis Software (Malvern Instruments Ltd., Malvern, Worcestershire, UK) using one set of sites fitting model and the point‐to‐point method for subtraction of the control data.

### Steady‐state kinetics

Measurements of the initial velocity were performed for the NQO1 wild‐type and P187S variant in the presence and absence of BPPSA by using a Specord 200 plus spectrophotometer (Analytik Jena) at 25 °C. The assays were performed in 50 mm HEPES containing 150 mm NaCl, pH 7.0, with NADH as electron donor and menadione as electron acceptor. For all components, the concentrations were determined spectrophotometrically. The reaction mixture for the assay performed with variation of the concentration of NADH contained 2.5 nm NQO1 WT or 20 nm NQO1 P187S variant, 200 μm menadione (ε_333 nm_ = 2450 m ^−1·^cm^−1^, prepared in EtOH with a final concentration of 1% v/v), 1–10 mm NADH (ε_340 nm_ = 6220 m ^−1·^cm^−1^), and 10 times the *K*_D_ of FAD equal to 0.6 μm for NQO1 WT and 4.2 μm for the NQO1 P187S variant, respectively. For the assay with variation of the concentration of menadione, the reaction mixture consisted of 1 nm NQO1 WT or 5 nm NQO1 P187S variant, 10 mm NADH, 45 μm cytochrome C, and 2.5–40 μm menadione in the case of NQO1 WT and 5–60 μm in the case of NQO1 P187S variant (dissolved in EtOH with a final concentration of 1% v/v) and 0.6 and 4.2 μm of FAD for wild‐type and P187S variant, respectively. The reaction mixtures were incubated for 2 min at 25 °C and the reaction was initiated by addition of the enzyme, and the decrease in absorption of NADH was measured at 400 nm (determined for these measurements) in the case of variation of NADH and the increase at 555 nm due to the reduction of cytochrome C in the case of variation of menadione. The slope corresponding to the first 20 s was used for the analysis for all conditions. The kinetic parameters were determined using the graphpad prism 5.01 software for Windows (La Jolla, CA, USA).

### Inhibition and activation assays

The inhibiting and activating effects of BPPSA on NQO1 WT and NQO1 P187S variant were investigated by using a Specord 200 plus spectrophotometer (Analytik Jena) at 25 °C. The concentrations for the used components were all determined spectrophotometrically. The final reaction mixture contained 500 μm NADH (ε_340 nm_ = 6220 m ^−1·^cm^−1^), 200 μm menadione (ε_333 nm_ = 2450 m ^−1·^cm^−1^, dissolved in EtOH), 10 nm NQO1 WT or 20 nm NQO1 P187S variant, and 0–316 μm BPPSA (dissolved in EtOH) for the inhibition assay and 0–1600 nm BPPSA for the activation assay, respectively. In the case of NQO1 P187S variant, a final concentration of 4.2 μm FAD was included in the assay. The reaction mixtures were first incubated for 2 min at 25 °C, whereafter the reaction was started by addition of enzyme. For the activation assay, BPPSA was coincubated with the protein for at least 30 min before the reaction was started. The decrease in absorption caused by the breakdown of NADH was measured at 380 nm. For analysis, the slope corresponding to the first 30 s of the reaction was used. The IC_50_ values were calculated using the graphpad prism 5.01 software for Windows.

### Limited proteolysis

NQO1 WT and NQO1 P187S variant at a concentration of 15 μm in 50 mm HEPES, 150 mm NaCl, and pH 7.0 were digested with trypsin from Promega at a final concentration of 2 μg·mL^−1^ at 37 °C. Samples were prepared with protein (NQO1 WT or NQO1 P187S variant) and 30 μm FAD and with protein, 30 μm FAD, and 300 μm BPPSA. Trypsin was preincubated at 37 °C for 15 min where, after the digestion was started, samples were taken out after specific time points (0, 5, 10, 20, 40, 80, 160 min, and overnight for 18 h), and the reactions were stopped by addition of SDS sample buffer followed by boiling at 95 °C for 10 min. The samples were then analyzed by SDS/PAGE. 13, 24.

### MALDI‐TOF MS

The protein bands from the SDS/PAGE were cut out, washed, and destained. The protein samples in the gel were reduced by 10 mm dithiothreitol and the cysteines were alkylated by 55 mm iodoacetamide. The proteins in the gel were then digested with trypsin at 37 °C over night, whereafter peptides were extracted from the gel by using a buffer containing 50% acetonitrile and 5% formic acid. The extraction buffer was then evaporated in an ISS100 SpeedVac® system (Thermo Scientific, Waltham, MA, USA) for 2 h. The peptide mixtures were dissolved in double distilled water and 0.1% TFA and desalted with ZipTip (Millipore Merck, Burlington, MA, USA). The purified samples were spotted on a MALDI plate together with the matrix a‐cyano‐4‐hydroxycinnamic acid (10 mg·mL^−1^ in a 1 : 1 v/v ratio). After a washing step with cold double distilled water and 0.1% TFA, spectra were then recorded on a Micromass TofSpec 2E in reflectron mode with an accelerating voltage of +20 kV. A calibration of the instrument was done with a poly(ethylene glycol) mixture (Sigma‐Aldrich), and ProteoMass ACTH fragment 18–39 MALDI‐MS Standard (Sigma‐Aldrich) was used as the calibration standard for peptides. The spectra were analyzed with MASSLYNX 4.1 (Micromass UK Limited, Wilmslow, UK) and compared to peptide fragments predicted by PeptideMass 25.

### ^15^N‐Labeling of NQO1 WT and the NQO1 P187S variant

For the expression of the ^15^N‐labeled proteins, a minimal medium containing 6.8 g·L^−1^ Na_2_HPO_4_, 3 g·L^−1^ KH_2_PO_4_, 0.5 g·L^−1^ NaCl, 1 g·L^−1^ ^15^NH_4_Cl, 3 g·L^−1^ glucose, 1 μg·L^−1^ biotin, 1 μg·L^−1^ thiamine, 50 μg·mL^−1^ kanamycin, and 1 mL 1000× microsalts (150 mm CaCl_2_, 20 mm, FeCl_3_, 50 mm H_3_BO_3_, 150 μm CoCl_2_, 800 μm CuCl_2_, 1.5 mm ZnCl_2_, and 15 μm (NH_4_)_6_Mo_7_O_24_·4H_2_O) was used. Except for this difference in medium composition, the protein expression was carried out as described under the section Molecular cloning, heterologous protein expression, and purification of NQO1.

### NMR spectroscopy

For NMR acquisitions, ^15^N‐labeled NQO1 WT or NQO1 P187S variant was dialyzed into a HEPES buffer (50 mm, pH 6.5) to final concentrations of 300 μm NQO1 WT and 530 μm NQO1 P187S variant, respectively. To each sample, 10% ^2^H_2_O was added for field‐frequency locking. BPPSA was added to final concentrations of 440 μm in the case of NQO1 WT and 680 μm in the case of the NQO1 P187S variant. All spectra were recorded at 300 K on a Bruker Avance III 700 MHz NMR spectrometer, equipped with a 5 mm cryogenically cooled TCI probe with z‐axis gradients. Two‐dimensional ^1^H‐^15^N‐heteronuclear single quantum coherence (HSQCs) were acquired with data matrices of 2048 × 128 data points. Forty‐eight scans were recorded for each increment, and 60° phase‐shifted squared sine‐bell window functions were applied in both dimensions prior to Fourier transformation using TopSpin 3.1.

### Small‐angle X‐ray scattering

Small‐angle X‐ray scattering (SAXS) data for solutions of the bound or free ligand forms of NQO1 WT and P187S variant in the presence of the FAD cofactor were recorded on an in‐house SAXS instrument (SAXSpace, Anton Paar, Graz, Austria) equipped with a Kratky camera, a sealed X‐ray tube source, and a Mythen2 R 1K Detector (Dectris). The scattering patterns were measured with a 60‐min exposure time (60 frames, each 1 min) with a solute concentration of 200 μm. Radiation damage was excluded on the basis of a comparison of individual frames of the 60‐min exposures, wherein no changes were detected. A range of momentum transfer of 0.010 <s < 0.63 Å^−1^ was covered (s = 4π sin(θ)/λ, where 2θ is the scattering angle and λ is the X‐ray wavelength, in this case 1.5 Å).

All SAXS data were analyzed and processed using the SAXS analysis package (Anton Paar, version 3.0). The data were desmeared using GIFT 26. Forward scattering (I(0)), radius of gyration, (*R*_g_), maximum dimension (*D*_max_), and interatomic distance distribution function ((P(r)) were computed with GNOM 27. The masses of the solutes were evaluated based on their Porod volume.

### Hydrogen‐deuterium exchange coupled to mass spectrometry

A series of 2‐μL aliquots of 200 μm wild‐type NQO1 as well as the P187S variant, preincubated with 4.9 μm FAD, were diluted 1 : 20 with deuteration buffer (10 mm HEPES, pD 7.0, 150 mm NaCl, and 10 mm MgCl_2_). Analogous dilutions were prepared with wild‐type and variant samples preincubated with 750 μm BPPSA and 4.9 μm FAD to achieve the same final protein concentrations. Ligand concentrations in the labeling reaction are still 4.6‐ and 1.9‐fold above the *K*_D_ for the interaction of BPPSA with wild‐type NQO1 and the P187S variant, respectively.

Individual time points of the labeling reaction were sampled by removing 7.5 μL after 10, 60, 360, and 2400 s and immediate quenching with 52.5 μL ice‐cold buffer of 200 mm ammonium formate (pH 2.6) in the presence of 2.7 m urea. Samples were then injected into a cooled HPLC setup described in detail previously 28. Hydrogen‐deuterium exchange coupled to mass spectrometry (HDX‐MS) data were analyzed using the software package Hexicon 2 29.

### Crystallization and crystal structure determination

NQO1 WT was cocrystallized with BPPSA using the vapor diffusion method employing different commercial crystallization screens (Index, PEG/Ion from Hampton Research and Morpheus Screen from Molecular Dimensions). Drops were prepared by mixing 0.5 μL of the protein solution (at a concentration of 17 mg·mL^−1^ in 50 mm HEPES, pH 7.0, preincubated with a saturated solution of the ligand at a concentration of 750 μm) with an equal volume of mother liquor using an ORYX 8 pipetting robot (Douglas Instruments Ltd, Hungerford, Berkshire, UK). The trays were incubated at 20 °C. Yellow crystals were observed after approximately 1 month in various conditions. Diffracting crystals were obtained from the original condition A5 of the PEG/Ion screen consisting of 0.2 m magnesium chloride hexahydrate and 20% w/v polyethylene glycol 3350, pH 5.9.

Diffraction data were collected to a maximum resolution of 2.76 Å on beamline P11 (λ = 1.0089 Å) at DESY Hamburg, Germany. The crystals were trigonal (space group *P*3_1_12) with unit‐cell parameters *a* = *b* = 121.37 Å and *c* = 158.25 Å. The data were processed using iMOSFLM 30 and programs from the CCP4 suite 31. The structure was solved by molecular replacement using the program PHASER 32 with the structure of the NQO1 variant P187S (PDB code: http://www.rcsb.org/pdb/search/structidSearch.do?structureId=4CET) as a template. The structure was refined using the programs Coot 33 and PHENIX 34. Clear residual electron density was observed for FAD bound to each of the four chains in the asymmetric unit. Only in one of the four independent active sites (associated with chain B), the difference in electron density was clear enough to interpret it as a molecule of BPPSA in two alternate orientations. Detailed statistics pertaining to data processing and structure refinement are summarized in Table 1. Atomic coordinates and structure factors have been deposited in the Protein Data Bank under the accession number http://www.rcsb.org/pdb/search/structidSearch.do?structureId=6FY4.

**Table 1:** Data collection and refinement statistics for the crystal structure of NQO1 WT with BPPSA. Statistics for the highest‐resolution shell are shown in parentheses.

	NQO1 WT complex with BPPSA, PDB entry: http://www.rcsb.org/pdb/search/structidSearch.do?structureId=6FY4
Wavelength (Å)	1.009
Resolution range	52.75–2.76 (2.86–2.76)
Space group	*P*3_1_12
Unit cell (Å, °)	*a* = *b* = 121.37, *c* = 158.25 α = β = 90, γ = 120
Total reflections	641 366 (81 424)
Unique reflections	34 531 (4561)
Multiplicity	18.6 (17.9)
Completeness (%)	100 (100)
<*I*/σ(*I*)>	15.1 (5.3)
Wilson *B*‐factor (Å^2^)	49.0
*R*_merge_	0.158 (0.587)
*R*_meas_	0.162 (0.604)
*R*_pim_	0.038 (0.142)
CC_1/2_	0.996 (0.950)
Reflections used in refinement	34 483 (3393)
Reflections used for *R*_free_	1717 (139)
*R*_work_	0.2180 (0.3038)
*R*_free_	0.2903 (0.3797)
CC_work_	0.825 (0.810)
CC_free_	0.836 (0.745)
Number of non‐H atoms	8860
Macromolecules	8616
Ligands	244
Average *B*‐factor (Å^2^)	43.6
Macromolecules	43.8
Ligands	38.1
RMSD bonds (Å)	0.009
RMSD angles (°)	1.2
Ramachandran plot favored/allowed/outliers (%)	96.6/3.4/0.0
Rotamer outliers (%)	1.3
Clashscore	8.5

### Docking calculations

BPPSA was docked into the active site of NQO1 WT with the program ADFR 35 using 104 genetic‐algorithm optimization runs with a maximum of 2.5 million energy evaluations per run. The receptor was kept rigid, while the ligand BPPSA was flexible.

## Results

### Virtual and initial *in vitro* screening of possible stabilizing ligands

Initially, the goal of the virtual screening was to identify a small molecule that binds to the P187S variant protein and may exert a stabilizing effect. Toward this aim, the molecules in the ZINC database were docked and ranked according to their binding free energy. A subset, including eight of the best hits, was selected for an initial *in vitro* screening using the ThermoFAD method (Table S1), where BPPSA stood out as the ligand leading to the largest stabilizing effect, with an increase in melting temperature of 1 °C for the wild‐type and 2.5 °C for the NQO1 P187S variant. Because of the promising stabilizing effects of BPPSA, further experiments were performed investigating the effects of this compound on the stability and activity of NQO1 P187S variant as well as NQO1 WT.

### Binding and stabilizing effect of N‐(2‐bromophenyl)pyrrolidine‐1‐sulfonamide

**Figure 1:**
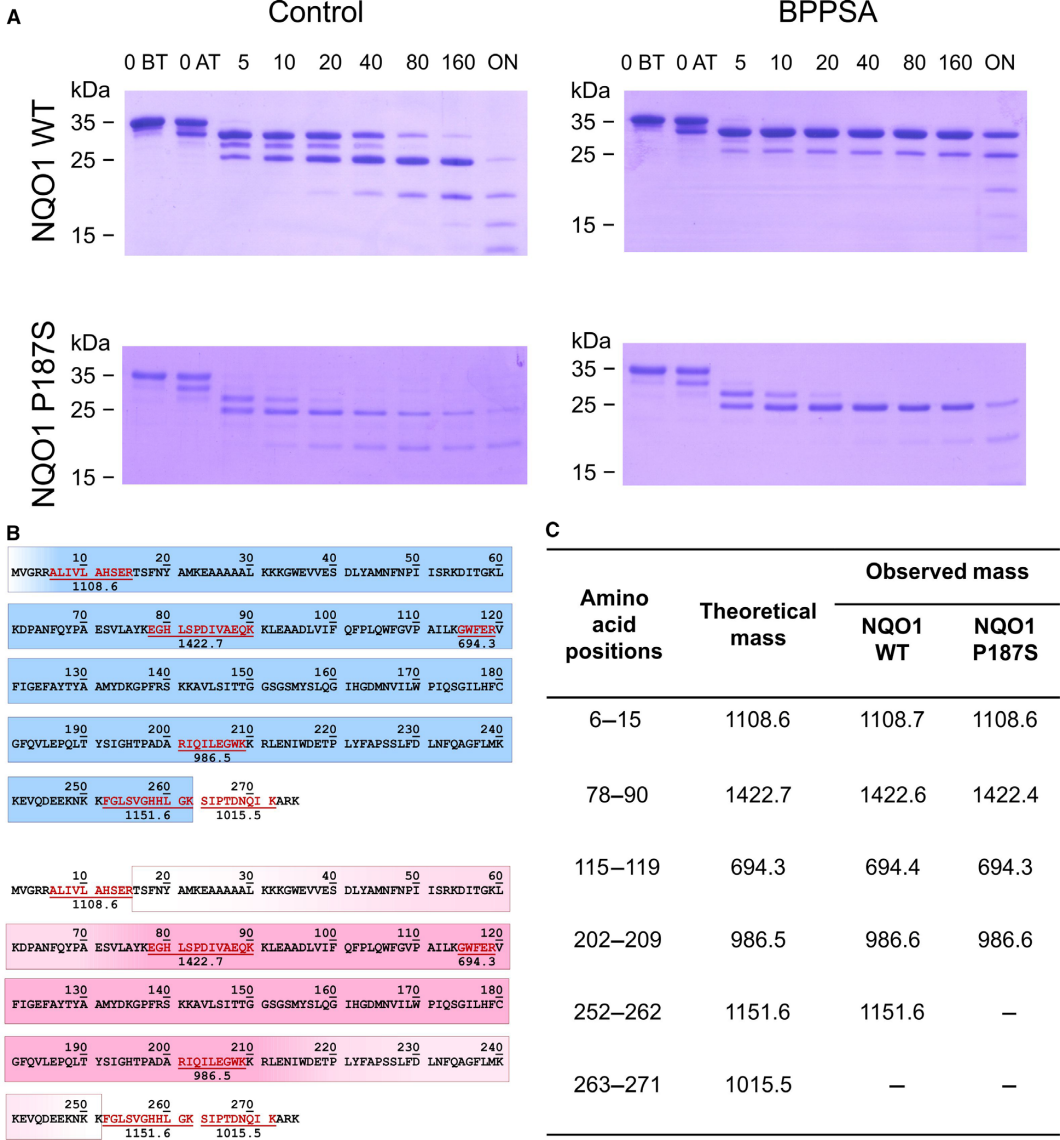
Limited trypsin proteolysis and MALDI‐TOF analysis of NQO1 WT and NQO1 P187S in the presence and absence of BPPSA. The compound stabilizes both the wild‐type and the P187S variant protein. (A) SDS/PAGE displaying the digestion over time for NQO1 WT and NQO1 P187S variant in the presence and absence of BPPSA. Lanes 1 and 2 show protein without trypsin (O BT) and protein taken out directly after addition of trypsin (O AT), lanes 3–8 show the digestion after 5–160 min, and the last lane shows the digestion overnight. (B) Theoretical fragments after trypsin digestion (displayed in red) were used to determine which part of NQO1 WT (blue) and NQO1 P187S variant (pink) was stabilized by the compound over time, and areas illustrated with a gradient could not be assigned due to too less information. (C) Peptides observed after MALDI‐TOF analysis of the stabilized SDS gel bands.

To further investigate how the ligand BPPSA influences the stability of NQO1, limited proteolysis experiments were performed in the presence and absence of BPPSA. In Fig. 1 the resulting SDS/PAGE analyses are displayed. Apparently, both the wild‐type and P187S variant proteins are partly stabilized in the presence of BPPSA. However, the stabilizing effect is different for the two proteins and correlates with the loss of peptides at the C terminus (Fig. 1C).

**Figure 2:**
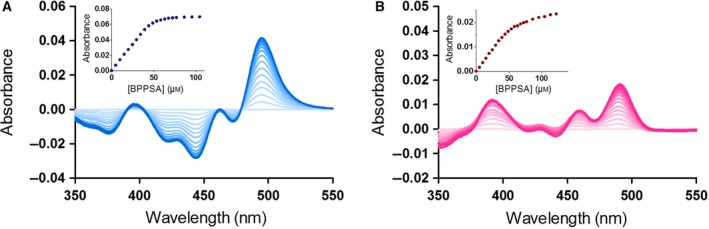
Difference absorption spectra of the ligand BPPSA titrated with NQO1 WT and NQO1 P187S. UV–Visible difference absorption spectra of (A) 40 μm NQO1 WT titrated with BPPSA (0–104 μm); the absorption spectra in the range from 350 nm to 550 nm are displayed in different shades of blue. The inset shows a plot of the change in the absolute absorption values at 444 and 496 nm against the concentration of BPPSA. (B) UV–Visible difference absorption spectra of 40 μm NQO1 P187S variant titrated with BPPSA (0–122 μm); the absorption spectra in the range from 350 to 550 nm are displayed in different shades of pink. The inset shows a plot of the change in the absolute absorption values at 440 nm and 491 nm against the concentration of BPPSA.

The binding of BPPSA to the wild‐type and the P187S variant could also be monitored using difference UV–Vis absorption spectroscopy. As shown in Fig. 2, titration of BPPSA to wild‐type protein produced a sharp endpoint, whereas the equivalent experiment with the P187S variant led to a biphasic binding behavior. In both cases, however, the absorption changes are similar and clearly indicate that binding of BPPSA affects the absorption properties of the FAD cofactor in the protein.

**Figure 3:**
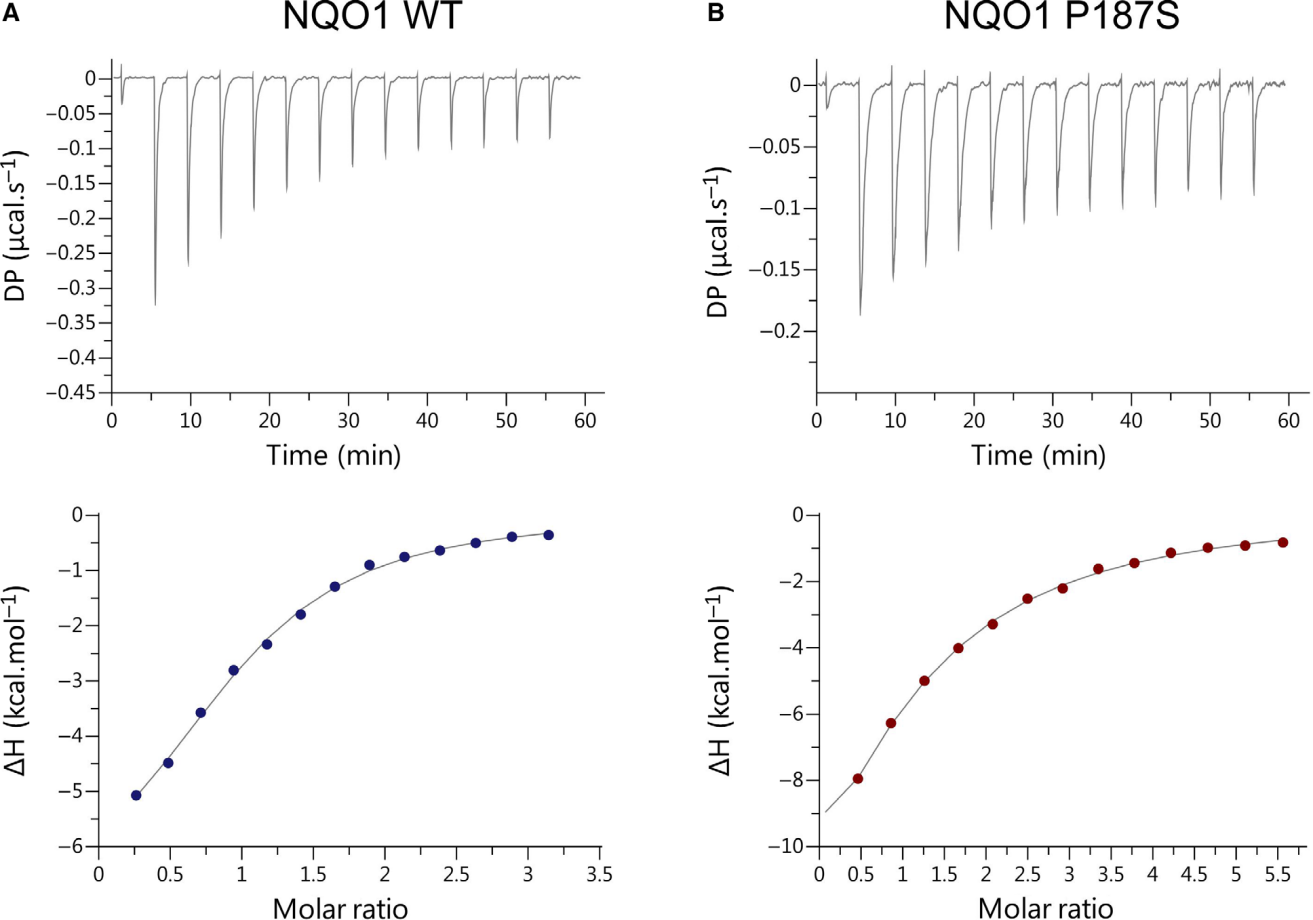
ITC measurements of BPPSA titrated with NQO1 WT or the NQO1 P187S variant. The dissociation constants for the binding of BPPSA to (A) NQO1 WT and (B) NQO1 P187S could be determined to be 7.95 ± 0.80 and 20.0 ± 0.41 μm, respectively. The experiments were performed in triplicates.

Next, the binding of the ligand was further investigated by ITC. The dissociation constants for the binding of BPPSA to the wild‐type and P187S variant could be determined to be 7.95 ± 0.80 and 20.0 ± 0.41 μm, respectively (Fig. 3 and Table S2).

### NMR spectroscopy, SAXS, and HDX‐MS measurements

**Figure 4:**
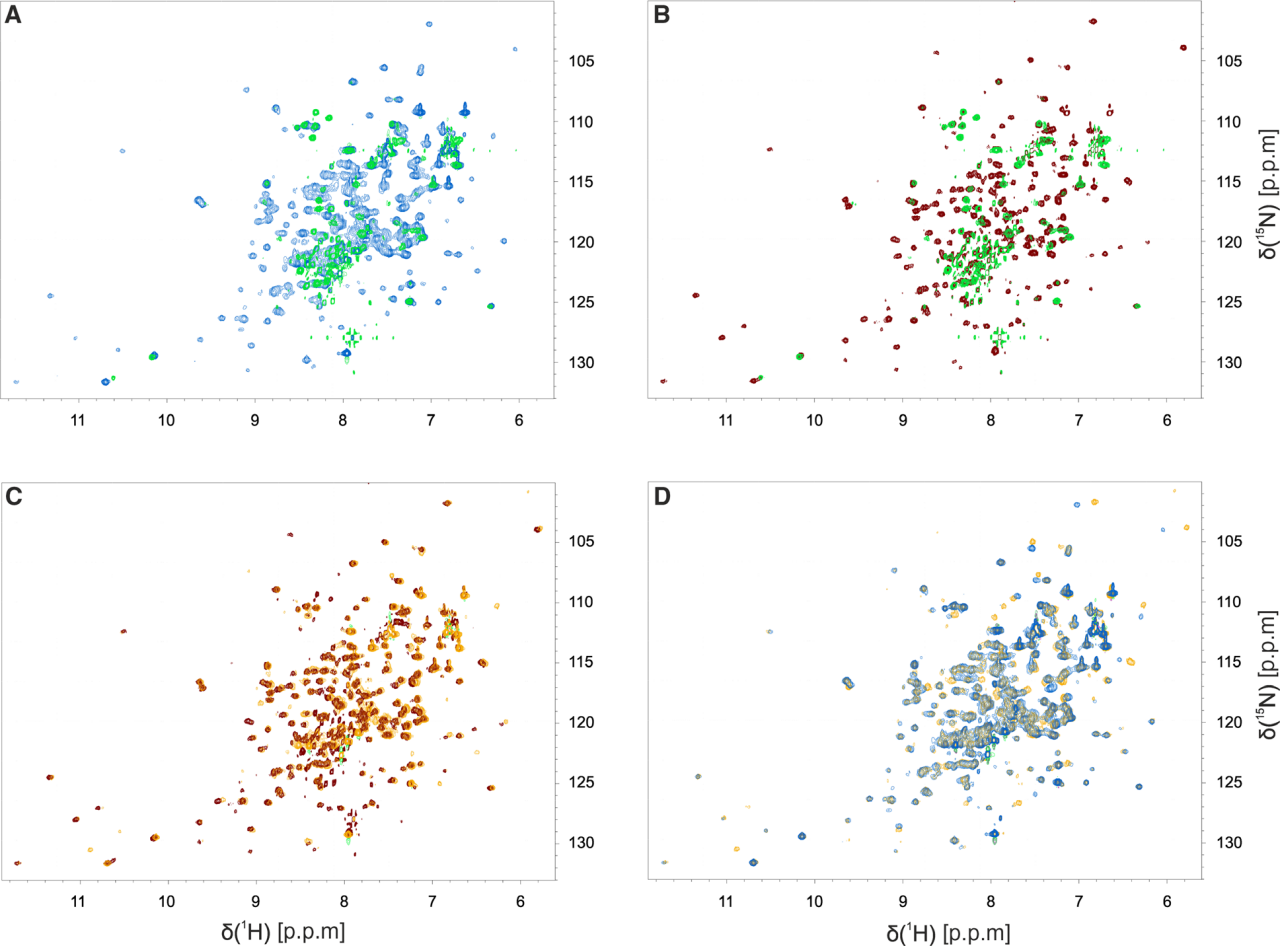
^1^H‐^15^N HSQC spectra of NQO1 WT and the P187S variant in the presence and absence of BPPSA. Overlay of ^1^H‐^15^N HSQC spectra of (A) the NQO1 P187S variant (green) and NQO1 WT (light blue), (B) the NQO1 P187S variant (green) and the NQO1 P187S variant with BPPSA (maroon), (C) the NQO1 P187S variant with BPPSA (maroon) and NQO1 WT with BPPSA (yellow), (D) NQO1 WT (light blue) and NQO1 WT with BPPSA (yellow).

To analyze the structural effects of BPPSA on the properties of the wild‐type and P187S variant protein in solution, we employed different biophysical methods that report on folding, shape, and accessibility of proteins. Recently, we have demonstrated that the ^1^H‐^15^N‐HSQC spectra of wild‐type NQO1 and the P187S variant exhibit significant differences: While the HSQC of the wild‐type enzyme is indicative of a relatively rigid and well‐structured protein, the P187S variant mainly showed signals in the random coil region, which likely result from increased flexibility of this variant (Fig. 4A) 13. Remarkably, the HSQC‐NMR spectrum for the NQO1 P187S variant improved in the presence of BPPSA indicating that the mobility of amino acid residues is substantially reduced (Fig. 4B). In fact, the HSQC spectrum of the P187S variant in the presence of BPPSA is similar to wild‐type NQO1 and virtually identical to wild‐type NQO1 in the presence of BPPSA, as shown in Fig. 4, panels C and D, respectively. This also confirmed that the ligand binds the wild‐type and the P187S variant at the same site.

**Figure 5:**
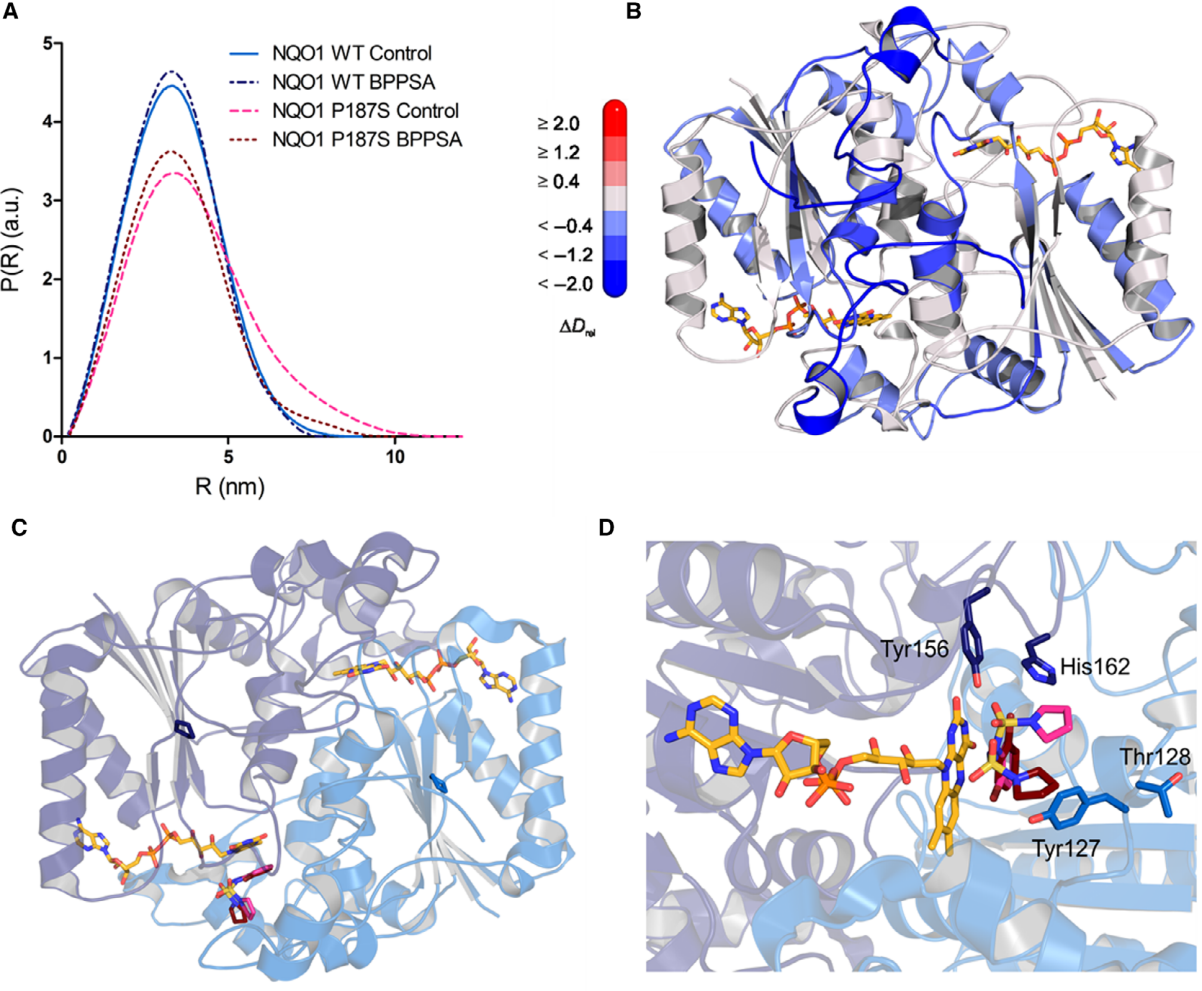
SAXS radial density distribution and conformational dynamics observed by HDX‐MS and crystal structure of NQO1 WT with docked BPPSA. (A) Small‐angle scattering measurement of NQO1 WT and the P187S variant in the presence and absence of BPPSA. The SAXS data displays a comparison of the experimental radial density distribution (P (R)) of NQO1 WT control (light blue), NQO1 WT with BPPSA (dark blue), the NQO1 P187S variant control (pink), and the NQO1 P187S variant with BPPSA (maroon). (B) Structure of the NQO1 P187S variant (PDB code: http://www.rcsb.org/pdb/search/structidSearch.do?structureId=4CET) with colors corresponding to the relative incorporation of deuterium, ∆*D*_rel_, between the NQO1 P187S variant in presence of BPPSA relative to the NQO1 P187S variant without BPPSA after 6 min of deuteration. The scale bar indicates the changes in ∆*D*_rel_ where blue corresponds to less incorporation of deuterium and red to higher incorporation. (C) and (D) Structure of NQO1 WT with BPPSA. (C) Overall structure of NQO1 WT with the 2 protomers colored in different shades of blue. The amino acid position P187 that is replaced with serine in the variant protein is highlighted. FAD is colored yellow and the ligand BPPSA is colored in pink and maroon. (D) Binding of BPPSA to the active site with an overlay of the two possible orientations illustrated in pink and maroon. The possible interacting residues Tyr‐127 and Thr‐128 are displayed with light blue stick representation and the catalytic residues Tyr‐156 and His‐162 are shown in dark blue 38.

SAXS measurements confirmed that both wild‐type and the P187S variant protein occur as dimers in solution. However, the P187S variant featured an increase in both radius of gyration (*R*_g_) and maximum distance (*D*_max_) compared to the wild‐type protein (*R*_g_ of 2.60 and 3.04 nm, *D*_max_ of 8 and 11 nm for wild‐type and the variant protein, respectively). This indicates a more extended conformation of the P187S variant dimer compared to the wild‐type protein, as similar observations have previously been reported 13, 36, 37. The addition of BPPSA led to a decrease in both *R*_g_ and *D*_max_ for the P187S variant, whereas no significant changes were observed for the wild‐type protein (*R*_g_ of 3.04 and 2.69 nm and *D*_max_ of 11 and 9 nm, for the P187S variant in the absence and presence of BPPSA, respectively). This indicates that the NQO1 P187S variant undergoes compaction toward the native conformation upon binding of BPPSA (Fig. 5A).

The conformational dynamics of the P187S variant were also investigated by HDX‐MS. This method provides information on secondary structure stability of the protein under different conditions, in our case in the presence and absence of BPPSA. As can be seen in Fig. 5B, BPPSA stabilized the flexible C‐terminal part of the P187S variant protein resulting in slower incorporation of deuterium.

### Crystal structure and docking

The stabilizing effect of BPPSA observed with the solution methods prompted us to further elucidate the binding site using X‐ray crystallography. Therefore, wild‐type NQO1 was cocrystallized with BPPSA, and the structure was determined to a resolution of 2.7 Å. As shown in Fig. 5C, the overall structure of the protein is identical to the previously described structures of wild‐type NQO1 and the P187S variant 13. The crystal structure contains four chains in the asymmetric unit forming two dimers. Each protomer exhibits a typical α/β‐fold with the FAD cofactor bound at the C‐terminal edge of the central β‐sheet (Fig. 5C,D). The active site of the enzyme near the isoalloxazine moiety of the cofactor is located at the dimer interface and is built up by amino acid residues from both chains 38.

**Figure 6:**
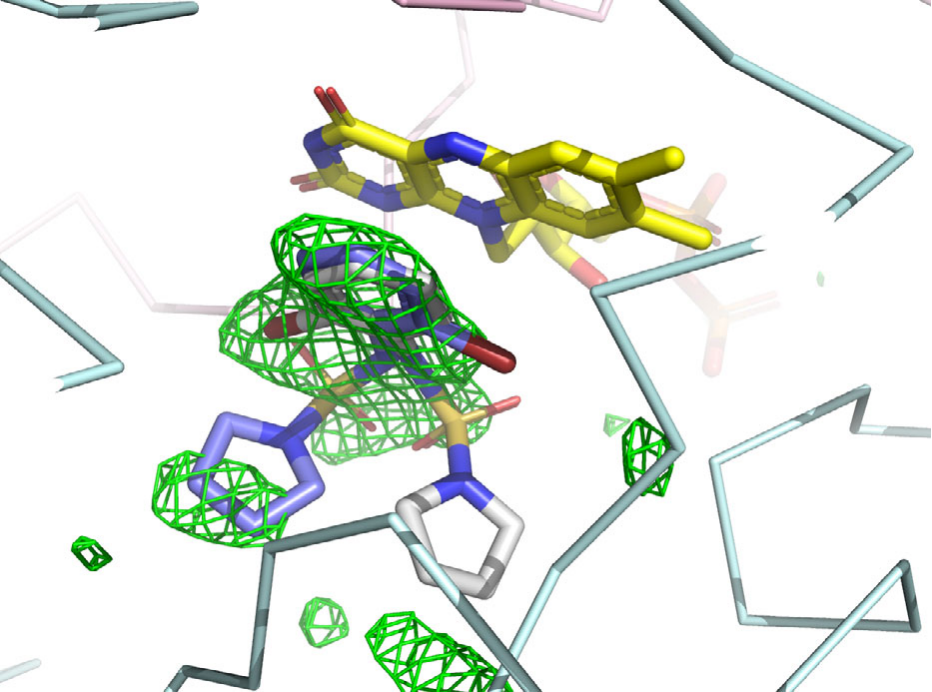
Polder map calculated for NQO1 WT. Polder map calculated in the active site of NQO1 WT and contoured at 3σ (green mesh). 39 The two binding modes of BPPSA, as well as the FAD cofactor, are shown in stick representations.

We observed residual electron density in one of the four crystallographically independent active sites. Although this density was rather weak (Fig. 6), we interpreted it as BPPSA bound to the active site of NQO1, consistent with binding modes of the ligand obtained by molecular docking calculations 39. In these binding modes, the bromophenyl group is stacked against the isoalloxazine ring (Fig. 5C,D) and is otherwise surrounded by mostly aromatic residues. This aromatic portion of the ligand is best represented by the residual electron density, whereas the position of the sulfonyl and pyrrolidine moieties is less well‐defined.

### Inhibition and activation assays

**Figure 7:**
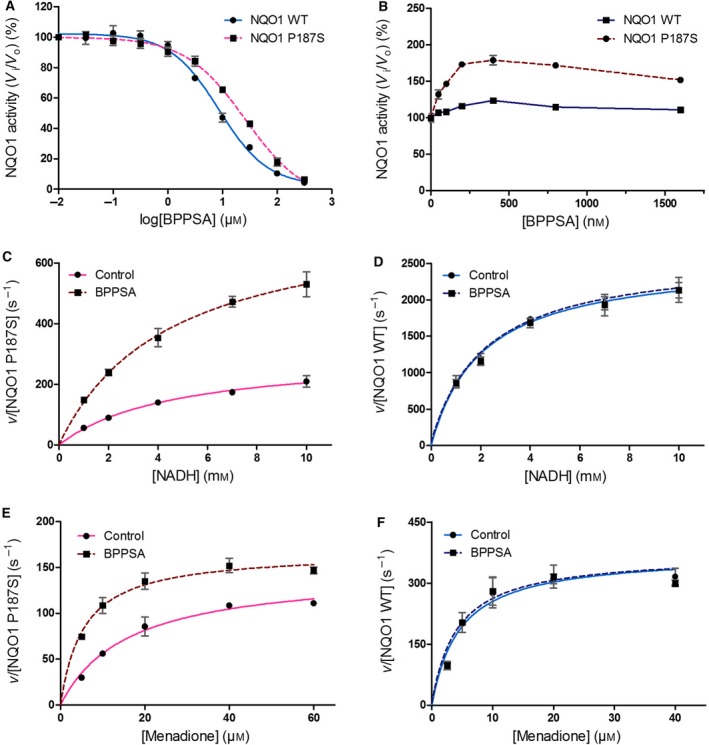
Inhibition, activation, and steady‐state kinetic assays for NQO1 WT and the P187S variant in the presence and absence of BPPSA. Inhibition by BPPSA is (A) shown for NQO1 WT (in blue) and the NQO1 P187S variant (in pink) and the corresponding IC_50_ values were calculated to be 8.6 ± 1.1 μm for NQO1 WT and  26.2 ± 1.2 μm for the NQO1 P187S variant. (B) Activation of NQO1 WT (dark blue) and the NQO1 P187S variant (maroon) in the presence of BPPSA. The velocity *v* over the enzyme concentration is plotted against the concentration of NADH for (C) the NQO1 P187S variant and (D) NQO1 WT and against the concentration of menadione for (E) the NQO1 P187S variant and (F) NQO1 WT.

Since the crystal structure together with the HDX‐MS measurements indicated that BPPSA binds to the active site, it was of interest to find out whether it acts as an inhibitor. From inhibition assays without coincubation with BPPSA and NQO1, IC_50_ values were calculated from the inhibition curves displayed in Fig. 7A, which was found to be 8.6 ± 1.1 and 26.2 ± 1.2 μm for wild‐type NQO1 and the P187S variant, respectively. However, if the protein is incubated with a low, noninhibitory concentration of BPPSA, an activation effect can be observed, with an increase in activity, especially, for the P187S variant as can be seen in Fig. 7B. The largest activating effect, leading to an 80% increase in activity for the P187S variant protein, could be observed at a final concentration of 400 nm BPPSA. Under the same conditions, only a minor increase in activity could be observed for the wild‐type protein.

### Steady‐state kinetics

Based on the activation assay described in the previous section, the concentration of BPPSA that exhibited the largest increase in activity (400 nm) was selected, and steady‐state kinetic parameters were determined for the wild‐type and P187S variant protein. As can be seen in Fig. 7 (panels C–F) and Table 2, the presence of BPPSA resulted in an increase in *k*_cat_/*K*_M_ for both NADH and the quinone substrate menadione in the case of the P187S variant protein. Under the same conditions, no substantial change in activity was observed for the wild‐type protein.

**Table 2:** Steady‐state kinetic parameters determined for NQO1 WT and NQO1 P187S variant with and without BPPSA. Kinetic parameters with standard errors were determined in triplicates for NQO1 WT and NQO1 P187S variant, using NADH as electron donor and menadione as electron acceptor and in the presence and absence of BPPSA.

	*k*_cat,app_ (s^−1^)	NADH *K*_M,app_ (mm)	*k*_cat,app_/*K*_M,app_ (mm ^−1^·s^−1^)	*k*_cat_ (s^−1^)	Menadione *K*_M_ (μm)	*k*_cat_/*K*_M_ (μm ^−1^·s^−1^)
NQO1 P187S						
Ctrl	300 ± 20	5 ± 1	70 ± 10	150 ± 7	16 ± 2	9 ± 1
BPPSA	770 ± 40	4 ± 0.5	170 ± 20	170 ± 4	6 ± 1	30 ± 4
NQO1 WT						
Ctrl	2550 ± 70	2 ± 0.2	1250 ± 110	370 ± 20	5 ± 1	80 ± 16
BPPSA	2580 ± 90	2 ± 0.5	1180 ± 140	370 ± 23	4 ± 1	90 ± 20

## Discussion

In the present study, we performed virtual screening with the aim to identify ligands that might have stabilizing effects on the structural instability of the NQO1 variant P187S. The shortlisted hits were further analyzed by *in vitro* screening experiments including thermal stability assays, absorption difference titrations, limited proteolysis, and ITC. These experiments identified BPPSA as the ligand with the best stabilizing properties. Interestingly, both the wild‐type and P187S variant structures were stabilized, however, to a different degree, as was observed in limited proteolysis experiments, indicating that a larger portion of the C‐terminal domain was stabilized in the wild‐type compared to the P187S variant (Fig. 1). The more pronounced effect of BPPSA (note that a saturating concentration of BPPSA, i.e., 2 mm, was used in the experiments) on the thermal stability of the P187S variant indicates that a larger fraction of the variant protein is in a (partially) unfolded state as compared to wild‐type NQO1.

Due to the interesting effect on the protein stability, further analyses of the stabilizing effect of BPPSA were performed using more advanced techniques, such as NMR, HDX‐MS, and SAXS. Based on our HSQC‐NMR data, it was observed that the P187S variant protein in the presence of BPPSA displayed an increased spectral dispersion, most probably due to reduced flexibility of residues in the P187S variant protein. The P187S variant also exhibited a more compact conformation, similar to that of the wild‐type, in the presence of BPPSA as shown by SAXS measurements. Finally, HDX‐MS experiments revealed in detail that the flexible C‐terminal domain of the protein was stabilized. Consequently, these experiments have illustrated that the P187S variant in the presence of BPPSA repopulates a conformation similar to the wild‐type, in particular with regard to the C‐terminal domain. This is consistent with other data showing that the C terminus is largely destabilized in the P187S variant, leading to the loss of the FAD cofactor and consequently of the enzymatic activity 13.

The determination of the crystal structure further demonstrated that BPPSA binds to the active site, where it seems to interact with the residues Tyr‐127 and Thr‐128. Since binding of BPPSA in or near the active site could potentially compromise the activity of the enzyme, the possible inhibitory effect as well as steady‐state kinetic parameters were determined, showing that coincubation of BPPSA with the enzyme (in the presence of excess flavin cofactor) leads to a substantial increase in catalytic efficiency of the P187S variant protein. Hence, under ideal conditions, it is possible that BPPSA can be used to rescue the enzymatic activity *in vitro*. The most probable reason for the advantageous effect on the activity seems to be connected to a higher loading of FAD cofactor to the P187S variant protein, consequently resulting in a higher population of holoenzyme present during the reaction. This also explains the minor differences observed in the activity of the wild‐type enzyme, since the FAD cofactor was reported to bind approximately 7 times tighter as compared to the P187S variant protein 13.

In the present study, we have demonstrated the potential of a small molecule to rescue the stability of a naturally occurring variant of NQO1 with beneficial effects on enzymatic activity. The binding and stabilization by small chemical chaperones may, in fact, provide a general concept to rescue intrinsically unstable human protein variants, for example, in cases where SNPs lead to structural instability. This concept may also be applicable to many other adverse processes that involve dysregulation of protein homeostasis, for example, in diseases that are related to protein aggregation. There are already examples available where pharmacological chaperones are studied or in use as treatment. One example is the treatment of the lysosomal storage disorder in Fabry disease with the chaperone migalastat that stabilizes the misfolded protein α‐galactosidase A during the transport from the endoplasmic reticulum to the lysosome 16, 17, 18. Another example is alpha‐1 antitrypsin deficiency, where polymerization of alpha‐1 antitrypsin possibly could be prevented by a chemical chaperone 19, 20. Thus, chemical chaperones may prove very useful as a complement in therapeutic intervention strategies targeting diseases associated with protein instability.

## Author contributions

W‐DL and PM initiated the project; ES, W‐DL, AH‐J, KZ, KG, and PM designed the experiments and analyzed the data; ES and W‐DL expressed and purified NQO1; AH‐J and KG crystallized NQO1 and determined the crystal structure; ES, W‐DL, AH, and DW performed analytical and biochemical experiments, and determined dissociation constants and kinetic parameters; AW performed and interpreted HDX‐MS measurements; KZ performed and interpreted NMR‐experiments; BB and TM performed and interpreted SAXS measurements; ES, W‐DL, BB, AW, KZ, KG, and PM wrote the manuscript.

## Supporting information


**Table S1.** Changes in thermal stability observed for NQO1 P187S during the initial experimental screening of ligands found by virtual screening.
**Table S2.** Thermodynamic parameters obtained through ITC measurements of the binding of BPPSA to NQO1 P187S and NQO1 WT respectively.
