# Supplementary material for: A small molecule chaperone rescues the stability and activity of a cancer‐associated variant of NAD(P)H:quinone oxidoreductase 1 in vitro
Source: FEBS Lett. 2019 Oct 30;594(3):424–38. doi: 10.1002/1873-3468.13636 (PMC7027498; doi:10.1002/1873-3468.13636)
Supplement: Supplementary file 1 — Table S1. Changes in thermal stability observed for NQO1 P187S during the initial experimental screening of ligands found by virtual screening. Table S2. Thermodynamic parameters obtained through ITC measurements of the binding of BPPSA to NQO1 P187S and NQO1 WT respectively. [file FEB2-594-424-s001.docx]

**Supplementary Information**

**Table S1.** Changes in thermal stability observed for NQO1 P187S during the initial experimental screening of ligands found by virtual screening.

| Ligand | Δ*T_m_* (°C) |
| --- | --- |
| (3-((butyl(methyl)ammonio)methyl)benzoyl)-L-prolinate | 0.3 |
| 1-[benzyl(methyl)sulfamoyl]pyrrolidine-2-carboxamide | -0.2 |
| N-cyclopropyl-1-(dimethylsulfamoyl)pyrrolidine-2-carboxamide | 0.4 |
| N-cyclopropyl-1-[[(3R)-3-methyl-1-piperidyl]sulfonyl]pyrrolidine-2-carboxamide | 0 |
| N-cyclopropyl-1-(1-piperidylsulfonyl)pyrrolidine-2-carboxamide | -0.6 |
| N-cyclopropyl-1-(diethylsulfamoyl)pyrrolidine-2-carboxamide | 0 |
| 6-pyrazol-1-yl-N-[5,6,7,8-tetrahydroimidazo[1,2-a]pyridin-6-yl]pyridine-3-carboxamide | -0.1 |

**Table S2.** Thermodynamic parameters obtained through ITC measurements of the binding of BPPSA to NQO1 P187S and NQO1 WT respectively. All measurements were performed in triplicates and the errors are shown as standard errors for all parameters.

|  | N | *K_D_*  (µM) | Δ*G*  (kcal·mol^-1^) | Δ*H*  (kcal·mol^-1^) | *-T*Δ*S*  (kcal·mol^-1^) |
| --- | --- | --- | --- | --- | --- |
| NQO1 P187S | 0.97 ± 0.03 | 20.0 ± 0.41 | -6.41 ± 0.01 | -23.4 ± 0.09 | 17.0 ± 0.12 |
| NQO1 WT | 0.95 ± 0.06 | 7.95 ± 0.80 | -6.97 ± 0.06 | -7.48 ± 0.17 | 0.51 ± 0.13 |
